# Comprehensive analysis of lactylation-related gene and immune microenvironment in atrial fibrillation

**DOI:** 10.3389/fcvm.2025.1567310

**Published:** 2025-04-22

**Authors:** Yazhe Ma, Youcheng Wang, Yuanjia Ke, Qingyan Zhao, Jie Fan, Yang Chen

**Affiliations:** ^1^ Yunnan Arrhythmia Research Center, The First People's Hospital of Yunnan Province & The Affiliated Hospital of Kunming University of Science and Technology, Kunming, Yunnan, China; ^2^ Department of Cardiology, The Affiliated Dongguan Songshan Lake Central Hospital, Dongguan Key Laboratory of Cardiovascular Aging and Myocardial Regeneration, Dongguan Cardiovascular Research Institute, Dongguan, China; ^3^ Department of Cardiology, Renmin Hospital of Wuhan University, Wuhan, China; ^4^ Cardiovascular Research Institute, Wuhan University, Wuhan, China; ^5^ Hubei Key Laboratory of Cardiology, Wuhan, China; ^6^ Department of Pathology, The First People's Hospital of Yunnan Province, The Affiliated Hospital of Kunming University of Science and Technology, Kunming, Yunnan, China

**Keywords:** atrial fibrillation, lactylation, immune infiltration, machine learning, diagnostic biomarkers

## Abstract

**Background:**

Atrial fibrillation (AF) is a common arrhythmia associated with an increased risk of stroke, heart failure, and mortality. Immune infiltration plays a crucial role in AF pathogenesis, yet its mechanisms remain unclear. Lactylation, a novel post-translational modification, has been implicated in immune regulation, but its association with AF remains unexplored. This study aims to elucidate the relationship between lactylation and immune infiltration in AF and identify potential diagnostic biomarkers.

**Methods:**

Gene expression data from left atrial tissue samples of AF and sinus rhythm (SR) patients were obtained from the Gene Expression Omnibus (GEO) database (GSE41177, GSE79768, GSE115574, GSE2240, GSE14975, and GSE128188). Differentially expressed genes (DEGs) between AF and SR samples were identified, followed by pathway enrichment and immune infiltration analysis. Correlation analysis and WGCNA were performed to assess interactions between lactylation-related genes and immune-associated DEGs. Machine learning models, including Random Forest and Support Vector Machine (SVM), were applied to select potential AF-related diagnostic biomarkers, and validated in the animal model (beagles; *n* = 6).

**Results:**

A total of 5,648 DEGs were identified, including six lactylation-related genes (DDX39A, ARID3A, TKT, NUP50, G6PD, and VCAN). Co-expression and WGCNA analyses identified lactylation- and immune-associated gene modules in AF. Functional enrichment analysis highlighted immune-related pathways such as T cell activation and neutrophil degranulation. A five-gene diagnostic model (FOXK1, JAM3, LOC100288798, MCM4, and RCAN1) achieved high predictive accuracy (AUC = 0.969 in training, 0.907 in self-test, and 0.950, 0.760, 0.890 in independent datasets). Experimental validation confirmed the upregulated expression of these biomarkers in AF.

**Conclusion:**

This study reveals a strong association between lactylation-related genes and immune infiltration in AF, suggesting their involvement in immune remodeling. The identified five-gene signature serves as a potential diagnostic biomarker set, offering novel insights into AF pathogenesis and contributing to improved diagnosis and targeted therapeutic strategies. Future studies integrating proteomic and single-cell analyses will further clarify the role of lactylation in AF.

## Introduction

Atrial fibrillation (AF), as one of most common sustained arrhythmias, affects nearly 33.5 million adults worldwide (1, 2). AF have a markedly elevated risk of stroke, heart failure, cardiovascular hospitalization, and mortality (3). Although catheter ablation can effectively treat some patients, the long-term success rate is low, and the recurrence rate varies from 24% to 39.4% (4, 5). It is of great importance to explore AF-related mechanism for therapeutic innovation. It has been generally accepted that immune play an important role in the AF pathogenesis. Previous studies have found reduced levels of infiltration of activated mast cells and regulatory T cells (Tregs) and increased levels of infiltration of *γδ* T cells, resting mast cells, and M2 macrophages in patients with AF compared with individuals in sinus rhythm (SR) (6). These changes in the infiltration of immune cells in AF suggest that immune mechanisms play a key role in AF. However, more studies are needed to further fully explore the mechanisms of immune infiltration in AF and may help to discover some new immunotherapeutic approaches for AF.

Lactic acid was thought as one of intermediated product of glycolysis until protein lactylation was firstly reported in 2019 (7). Numerous studies has proved that lactylation is a crucial post-translational modification and associated with various of cardiovascular diseases such as ischemic heart diseases, heart failure, hypertension and atherosclerosis (8–10). Recent studies highlight that lactylation is involved in cellular metabolic reprogramming, macrophage depolarization, and tissue inflammation (11, 12). AF will experience a substantial change in energy metabolism, which means switching from lipid metabolism to glycolysis, and significantly increased atrial lactate production (13). However, the association between expression of lactylation-related gene, immune infiltration, and AF has not been reported yet.

In this study, we assessed the immune microenvironment explore the relationship between immune infiltration and lactylation in AF. Then, machine learning algorithm s were applied to filter the crucial genes which associated with lactylation in AF patients. The implementation of these efforts will help us gain insight into the pathogenesis of AF from another perspective.

## Methods

This study was approved by the animal studies subcommittee of our institutional review board (WDRM 20191211) and was in compliance with the guidelines of the National Institutes of Health for the care and uses of animal experiment.

### Data sources

Three datasets (GSE41177, *N* = 38; GSE79768, *N* = 13, left atrium; GSE115574, *N* = 29, left atrial) were obtained from the GEO database and used as a training set after removing the batch effect through *sva* R package. The training set contained 53 AF patients and 27 SR individuals (Supplementary Figure S1). In addition, we also downloaded 3 datasets each as an independent test set (Testing dataset1: GSE2240, *N* = 30; Testing dataset2: GSE14975, *N* = 10; Testing dataset3: GSE128188, *N* = 15). The FPKM values were used to quantify the expression profiles, which were then normalized using a log2-based transformation. All of these samples were obtained from the left atrium.

Otherwise, 22 lactylation genes (ARID3A, CCNA2, DDX39A, EHMT2, ABP5, G6PD, H2AX, HMGA1, KIF2C, MKI67, PFKP, PKM2, RACGAP1, RFC4, STMN1, TKT, EFNA3, VCAN, PLOD2, HBB, NUP50, and STC1) were obtained from Jiao et.al (14).

### Animal model preparation

6 male beagles (age 1 years old), weighing an average of 8.6 ± 1.2 kg, were used in this study. the sham group (*n* = 3) received pacemaker implantation without atrial pacing, and the AF group (*n* = 3) received pacemaker implantation with continuous 7-day atrial pacing. During the pacemaker implantation, each beagle was administered an intramuscular injection of 25 mg/kg ketamine sulfate before being premedicated with pentobarbital sodium (30 mg/kg intravenously), intubated, and ventilated with room air supplemented with oxygen by a respirator (MAO01746, Harvard Apparatus Holliston, USA). The detailed methods were documented in our previous study (15). The AF was defined as an irregular atrial rate >500 bpm lasting for more than 5 s. At the end of the 7-day pacing, the animals were euthanized by overdose anesthesia. The left atrium tissues were quickly removed from the heart then perfumed qPCR.

### Immune microenvironment analysis

*xcell* R pacakage was used to evaluate the abundance of 64 different types immune cells and stromal cell, including T-cells, B cells, NK cells, Monocytes, Macrophages, Neutrophils, and so on. The comparison of immune cell abundance between AF and SR groups was conducted to identify a distinguishing trait of AF patients for subsequent analysis.

### Differential expression analysis and WGCNA

The gene expression levels between the AF and SR samples were compared using the ‘limma’ package in R. The differentially expressed mRNAs (DEGs) were identified with an absolute log2 fold change ≥0.263 and an adjusted *P*-value < 0.05.

The correlation between lactation genes and DEGs was calculated by Pearson analysis, and only DEGs with an absolute value of correlation coefficient greater than 0.8 and *P* < 0.05 were screened as lactic acid session-associated DEGs (LacDEGs).

The co-expression network of LacDEGs in AF patients was built using the ‘WGCNA’ R package and the automatic network construction function. Hierarchical clustering was implied to filter the similar expression patterns of gene modules. The characteristics of abnormal immune cells in AF patients were ultimately linked to these modules.

### Machine learning analysis

We used the randomForest R package to construct the AF prediction model, screening for combinations of genes that have a highly discriminatory efficacy for distinguishing between AF and SR group (assessing the relative importance of variables). Then, the most important genes were calculated through SVM algorithm based on the e1071 R package to construct the best diagnosis model.

### Function enrichment analysis

The study extracted all differentially expressed genes (DEGs) for additional functional enrichment using the Metascape webserver. Enrichment analysis was KEGG pathways, and Hallmarks. Functions with a false discovery rate < 0.05 was selected. GSEA analysis was performed through clusterProfiler R package.

### Statistical analysis

Statistical analyses were performed using R software (version 4.4.2). A *p*-value less than 0.05 was considered to be statistically significant.

### Quantitative real-time polymerase chain reaction (qPCR)

The expression of FOXK1, JAM3, LOC100288798, MCM4, and RCAN1 in left atium(LA) was measured by qPCR. Total RNA was isolated from LA samples with RNA Extraction Reagent (Vazyme, China) according to the manufacturer's protocol. cDNA was synthesized using EntiLink™ 1st Strand cDNA Synthesis Kit (ELK Biotechnology, China). The 2^–*ΔΔ*Ct^ method was used to calculate to the mRNA values of FOXK1, JAM3, LOC100288798, MCM4, and RCAN1. The primer sequences and amplicon sizes of the selected genes are shown in Table 1.

**Table 1:** Primer sequence of selected genes.

Gene	Primer	Sequence (5'-3’)	PCR products^a^
b-actin	Forward	CACGATGGAGGGGCCGGACTCATC	240bp
	Reverse	TAAAGACCTCTATGCCAACACAGT	
Dog FOXK1	Forward	ATTCCACACGACCCTGACTT	170bp
	Reverse	ACTATGGACGCTGGCATGTA	
Dog JAM3	Forward	CCGTGAATCTCAAGTCCAGC	198bp
	Reverse	TCCCCAATAGTTCTGCACGA	
Dog MCM4	Forward	ACTACCGGAGCGAAGAACAA	170bp
	Reverse	CATTCCTCGGCTGCTACCTA	
Dog RCAN1	Forward	CTTATCTGCAGCAGATGCCA	196bp
	Reverse	GACGGGAGTAGCATCTTCCA	
Dog LOC100288798	Forward	GGTTTGGGTCCTCCAATCCT	178bp
	Reverse	CAGTCATGCATGATGCCAGG	

^a^ bp, base pairs.

## Results

### Construction of the regulatory network related to lactylation genes in AF

**Figure 1:**
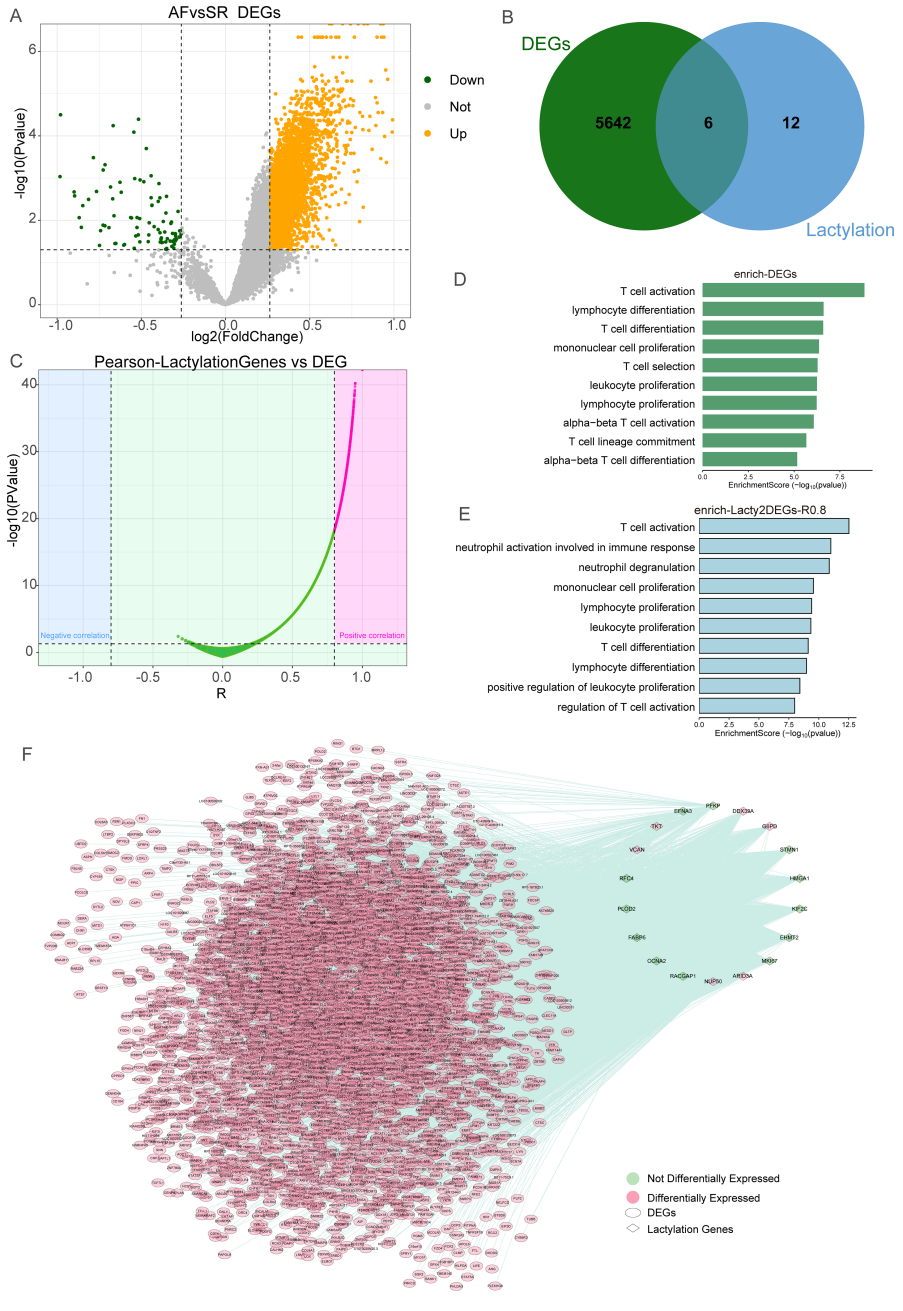
Construction of the lactylation gene regulatory network in AF. **(A)** Volcano plot showed the DEGs of AF vs. SR groups. **(B)** Venn plot showed that six lactylation genes were differentially expressed in AF. **(C)** The Pearson relationship between lactylation genes and DEGs in AF. **(D)** Enrichment analysis of DEGs in AF. **(E)** Enrichment analysis of DEGs associated with lactylation genes in AF. **(F)** Network of the Lactylation Gene and DEGs in AF.

After batch correction, we obtained the expression profile of 53 AF patients and 27 SR samples. We further screened the abnormally expressed genes in AF patients (Figure 1A), and a total of 5,648 differentially expressed genes (DEGs) were obtained. Then, we found that 6 lactylation genes (DDX39A, ARID3A, TKT, NUP50, G6PD, and VCAN) were also differentially expressed in AF (Figure 1B). Next, we performed Pearson correlation analysis of these DEGs with the 22 lactylation-related genes and screened 3,832 DEGs associated with lactylation (Figure 1C), interestingly, all the DEGs were positive associated with the lactylation-related genes. Enrichment analysis of the DEGs suggested that they were involved in T cell activation, T cell differentiation, and other immune-related pathways (Figure 1D). The lactylation-related DEGs also enriched in regulation of T cell activation, T cell activation, neutrophil degranulation, and other immune-related pathways (Figure 1E). These results suggest that this special immune microenvironment exists in AF. Then, we constructed the regulatory network related to lactylation genes in AF (Figure 1F).

### Assessment of the AF immune microenvironment and screening of lactylation-associated aberrant gene co-expression modules

**Figure 2:**
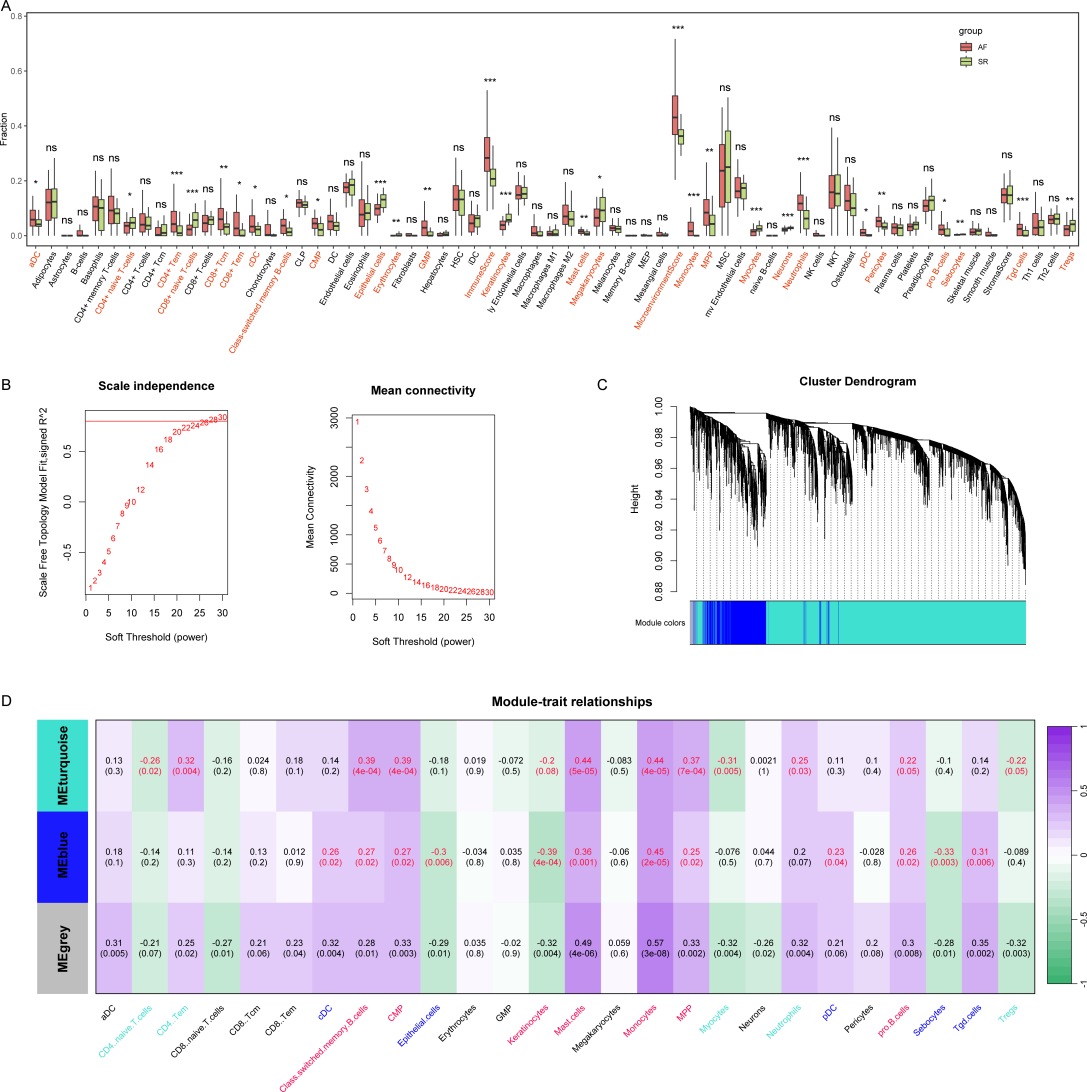
Comprehensive assessment of the immune microenvironment in AF and screening of immune cells associated co-expression modules in AF. **(A)** We used the xCell algorithm to assess the abundance of AF vs. SR groups. **(B)** The WGCNA analysis to screen co-expression modules, choose 28 as the power of the co-expression module. **(C)** Sample cluster of the co-expression modules. **(D)** Immune cells associated co-expression modules in AF.

Subsequently, we evaluated the immune microenvironment in these patients (Figure 2A), and the results showed that a variety of immune cells such as CD4^+^ naive T cells, CD8^+^ Tcm, and Monocytes were abnormal in AF patients. Next, we subjected these 3,832 DEGs to WGCNA analysis with 26 aberrant immune cells to obtain co-expression modules associated with these aberrant immune cells (Figures 2B,C**)**. The results showed that these DEGs had a total of 3 co-expression patterns (MEblue, MEturquoise and MEgrey), where MEblue and MEturquoise were associated with multiple immune cells (Figure 2D). The DEGs in the two modules were further used to select AF associated biomarkers.

### Identify AF-related diagnostic biomarkers

**Figure 3:**
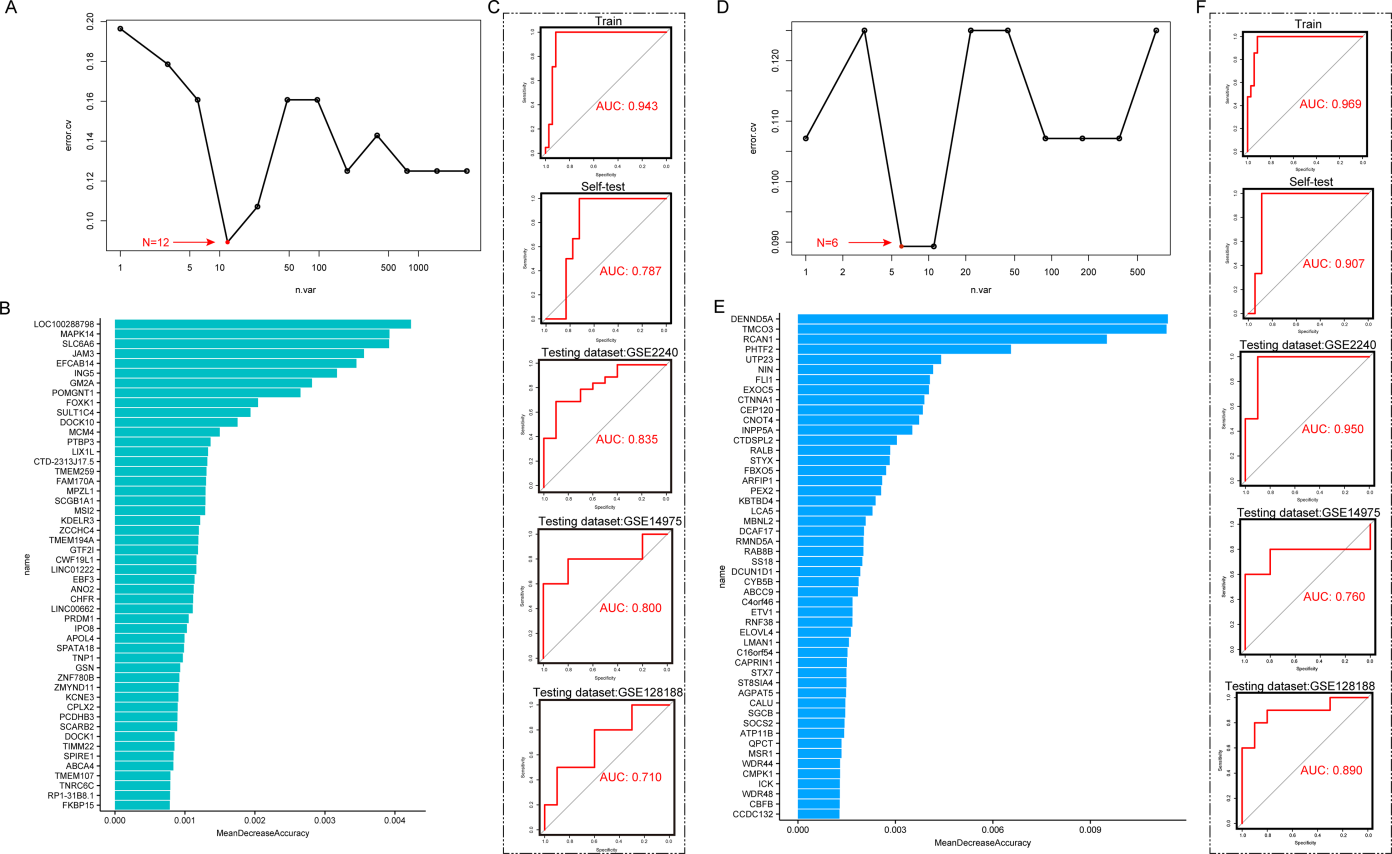
Screen diagnostic biomarkers through random forest algorithm in co-expression modules. **(A)** Random Forest analysis in MEturquoise group, the model gene number of 12 has the smallest error rate. **(B)** Mean Decrease Accuracy in the RF model of MEturquoise. **(C)** SVM performance of the RF model of MEturquoise in training dataset, self-test dataset and independent testing datasets. **(D)** Random Forest analysis in MEblue group, the model gene number of 6 has the smallest error rate. **(E)** Mean Decrease Accuracy in the RF model of MEblue. **(F)** SVM performance of the RF model of MEblue in training dataset, self-test dataset and independent testing datasets.

We then screened the genes in the MEturquoise and MEblue modules for AF-associated diagnostic markers based on Random Forest Algorithm. The MEturquoise module contained a total of 3,072 lactylation-associated DEGs, and the Random Forest Algorithm found that the model consisting of 12 DEGs had the smallest error rate (Figure 3A). Subsequently, we ranked the gene importance scores of the random forest (Figure 3B) and filtered the top 12 key genes to construct the model. The results showed that the diagnostic efficacy of this 12-gene model for AF in the training set reached 0.943, and the AUC in the self-test set reached 0.787. Subsequently, we validated the efficacy of this model in three independent testing datasets (Figure 3C, AUC_Testing dataset:GSE2240_ = 0.835, AUC_Testing dataset:GSE14975_ = 0.800, AUC_Testing dataset:GSE128188_ = 0.710**)**.

In the MEblue module, we also screened for AF-related diagnostic markers. The MEBlue module contained a total of 710 lactylation-associated DEGs, and the Random Forest algorithm found that a model composed of 6 DEGs had the smallest error rate (Figure 3D). Subsequently, we ranked the gene importance scoring of the random forest and filtered the top 6 key genes to construct the model (Figure 3E). The results showed that this 6-gene model achieved a diagnostic efficacy of 0.969 for AF in the training dataset, and an AUC of 0.907 in its self-test set. Subsequently, we validated the efficacy of this model in three independent test sets (Figure 3F, AUC_Testing dataset:GSE2240_ = 0.950, AUC_Testing dataset:GSE14975_ = 0.760, AUC_Testing dataset:GSE128188_ = 0.890).

### Constructed the optimal diagnostic model for AF

**Figure 4:**
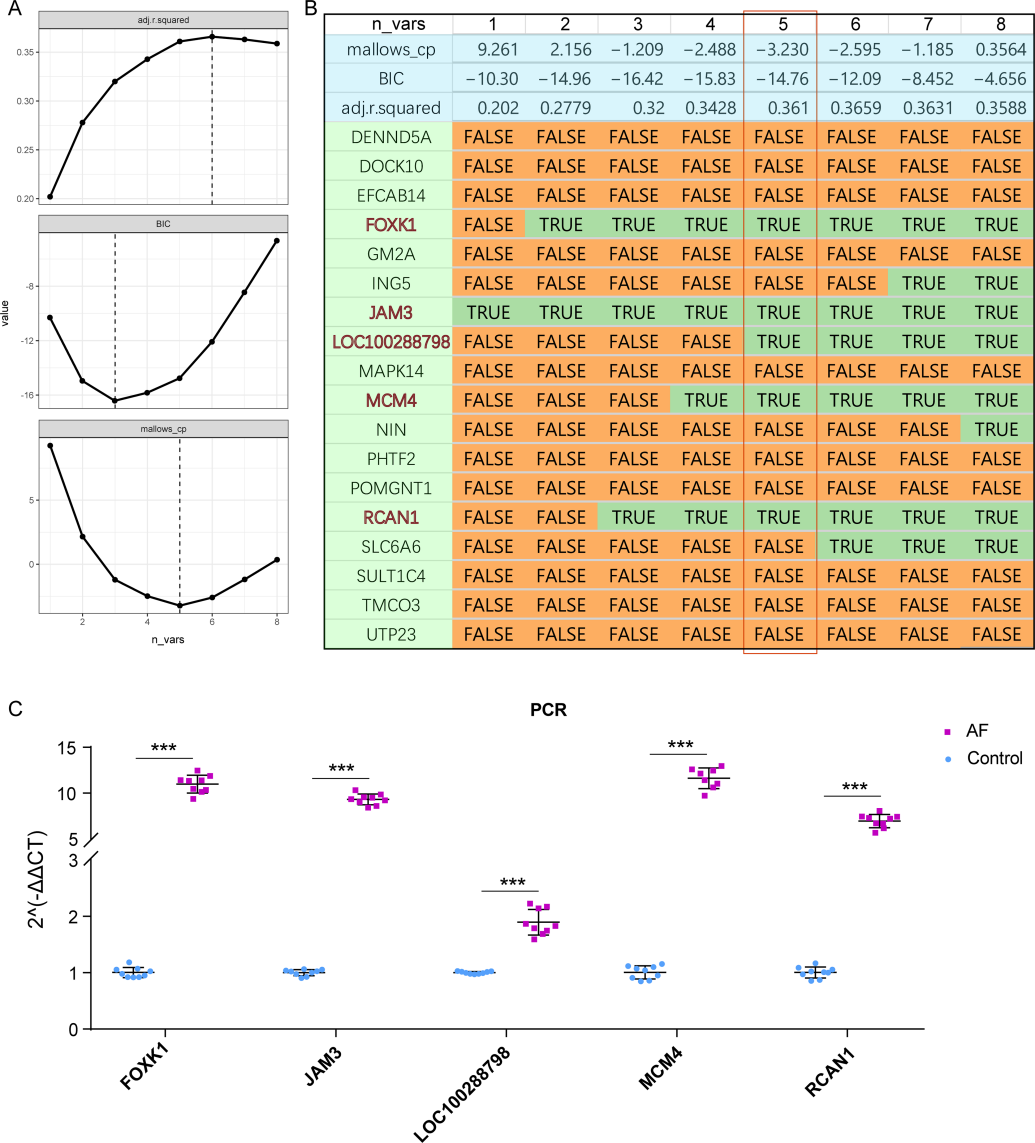
Constructing the AF diagnostic model. **(A)** Parameters of Best Subset Selection regression model, including Mallows_up, adjusted R2 and BIC. **(B)** Final diagnostic biomarkers selected by Best Subset Selection. Here, the five genes model has the best efficacy. **(C)** qRT-PCR of these five crucial genes in AF.

We then integrated the 12 MEturquoise diagnostic DEGs and 6 MEblue diagnostic DEGs and screened the optimal diagnostic model for AF based on the optimal subset method. Ultimately, we found that among these 18 candidate genes, a 5-gene model (FOXK1, JAM3, LOC100288798, MCM4, and RCAN1) demonstrated smaller BIC and mallow up values and higher R^2^ (Figure 4A), suggesting that this 5-gene model has better diagnostic efficacy in AF (Figure 4B). Finally, we validated the expression of these 5 key biomarkers in 12 adult beagles and showed that the expression of all five genes was significantly increased in AF (Figure 4C). This also suggests that the 5 biomarkers we screened have potential diagnostic efficacy in AF.

**Figure 5:**
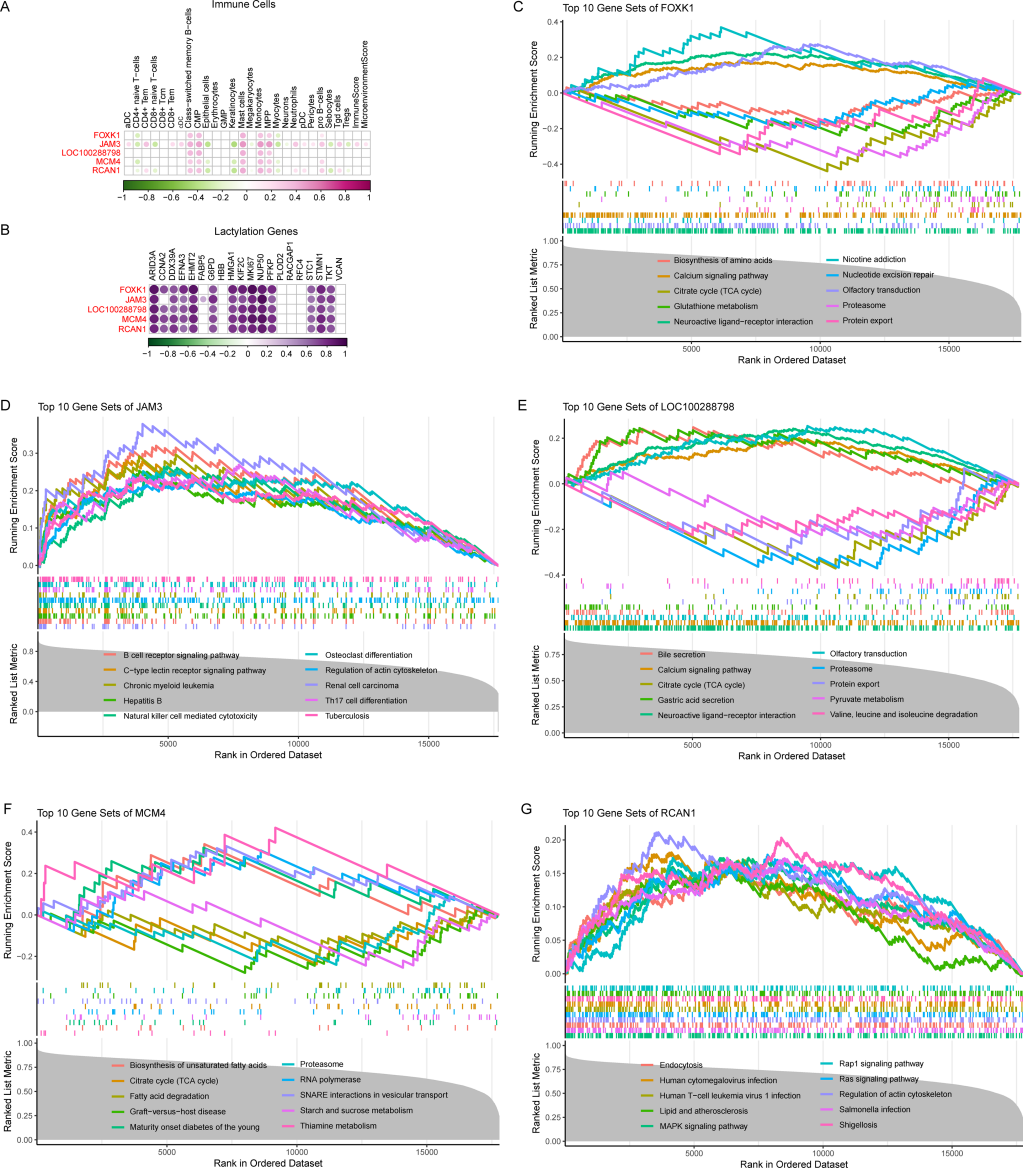
Comprehensive assessment of the five crucial genes in AF. **(A)** Pearson analysis between the five genes and abnormal immune cells in AF. **(B)** Pearson analysis between the five genes and lactylation genes in AF. **(C)** GSEA analysis of FOXK1 in AF. **(D)** GSEA analysis of JAM3 in AF. **(E)** GSEA analysis of LOC100288798 in AF. **(F)** GSEA analysis of MCM4 in AF. **(G)** GSEA analysis of RCAN1 in AF.

We further evaluated the correlation of these five genes with abnormal immune cells in AF. The results showed that all of them were associated with abnormal immune cells in AF, suggesting that they play a key role in the immune microenvironment of AF (Figure 5A). We then also correlated these 5 genes with lactate-associated genes, and the results showed that they were strongly positively correlated with most of the lactylation genes, further supporting that lactonization does have a potential role in AF (Figure 5B).

In conclusion, the five crucial biomarkers we screened are not only associated with lactylation and the immune microenvironment, but also have efficacy in the diagnosis of AF.

## Discussion

AF encompasses a series of pathogenesis changes, such as electrical remodeling, structural remodeling, and immune remodeling. It is accepted that AF development and maintenance is associated with recruitment and activation of immune cells in atria. There are studies on the effects of intracellular calcium overload, oxidative stress and cell apoptosis on the atrial immunity, however, it is still elusive on the effects of atrial metabolite, lactation-related gene on atrial immunity. To our knowledge, this is the first study to explore the relationship between immune infiltration and lactylation-related gene in AF.

Here, we identifying five crucial genes such as FOXK1, JAM3, LOC100288798, MCM4, and RCAN1 play an important role in atrial remodeling in AF. FOXK1, the transcriptional factor forkhead box K1, is involved in multiple process such as metabolism, proliferation, cell signaling, apoptosis, and DNA damage (16). Recent studies found FOXK1 is glycolysis regulator that contributes to energy metabolism shift from lipid to glycolysis (17). FOXK1 regulate CCL2 expression and recruit macrophages (18). In this study, we performed GSEA analysis and found that in AF patients, FOXK1 is involved in pathways such as the citric acid cycle (TCA cycle), glutathione metabolism, and calcium signaling pathway, where the citric acid cycle (TCA cycle) is closely related to lactate (Figure 5C). JAM3, a member of junction adhesion molecular family, is associated with hypoplastic left heart and extracellular matrix organization (19). Study also revealed that JAM3 is positively associated with infiltration of mast cell and M2 macrophage (20). Here, we found that JAM3 is associated with immune-related pathways such as the B cell receptor signaling pathway, Natural killer cell mediated cytotoxicity, and Th17 cell differentiation in AF (Figure 5D). LOC100288798 is also refered to SLC38A4 antisense RNA (SLC38A4-AS1), for the first time in this study, it was found to be associated with AF, and we also found that in AF, it was associated with metabolic and lactylation-related pathways such as the citric acid cycle (TCA cycle), calcium signaling pathway, protein export and pyruvate metabolism (Figure 5E). MCM4, minichromosome maintenance complex component 4, is important in DNA replication by acting as replication initiation factor and helicase (21). MCM4 is associated with immune infiltration especially for NKT cell (22), however, we found it also negative related to CD4 + naive T cells, Epithelial cells, Myocytes and positive related to B cell, CMP, Mast cells, Monocytes, and MPP in our study (Figure 5A). And it also been found to be associated with the citric acid cycle (TCA cycle), Fatty acid degradation, and Starch and sucrose metabolism (Figure 5F). RCAN1, regulator of calcineurin 1, functions in multiple process such as development and maintenance of cardiovascular system. Previous studies show that RCAN1 mutation can lead to congenital heart disease, and RCAN1 dysregulation contributes to cardiovascular diseases such as atherosclerosis, myocardial hypertrophy. Here, we found it is associated with Lipid and atherosclerosis, MAPK signaling pathway, Rap1 signaling pathway, Ras signaling pathway, and Regulation of actin cytoskeleton.

In this study, we analyzed gene expression data to identify key biomarkers associated with AF. However, gene expression alone does not fully capture the functional impact of these genes, as post-translational modifications (PTMs) play a crucial role in regulating protein activity, stability, and interactions. Among these, lactylation has been recognized as an important PTM influencing cellular metabolism, immune responses, and gene regulation. While our study does not directly assess lactylation modifications, it is possible that these genes identified in our analysis may be functionally regulated by lactylation. Future studies integrating proteomic and PTM-specific analyses will be necessary to determine the extent to which lactylation affects these proteins and their roles in AF.

Here, we primarily investigate the gene expression changes in the atrium, however, it is important to consider the potential role of immune cell infiltration in shaping these molecular signatures. Given that peripheral blood mononuclear cells (PBMCs) serve as a systemic indicator of immune activity, their gene expression patterns may provide valuable insights into the relationship between immune cells and AF. Previous studies have shown that immune signatures in PBMCs can reflect tissue-specific immune responses, suggesting that a comparative analysis between atrial gene expression and PBMC profiles could help elucidate the extent of immune infiltration (23). Studies incorporating single-cell RNA sequencing will help to elucidate the contribution of infiltrating immune cells in AF.

Furthermore, atrial fibrillation (AF) is characterized by disturbances in atrial electrophysiology, often driven by molecular and structural remodeling. The gene expression changes identified in our study may contribute to AF pathogenesis through several key mechanisms. Among them, FOXK1 and LOC100288798 are involved in ionic calcium (Ca^2+^) channel regulation, and alterations in calcium (Ca^2+^) channel expression may lead to action potential shortening and increased susceptibility to reentrant circuits, both of which are characteristic of AF. In addition, genes associated with fibrosis and structural remodeling may impair excitation propagation, thereby further contributing to the maintenance of AF. This suggests that the observed molecular alterations may be a link between AF and its associated risk factors. Future studies combining patient clinical data with transcriptome profiling could provide a more comprehensive understanding of these interactions.

In summary, we constructed a lactylation-related regulation network of AF and found that AF patients have a distinct immune microenvironment. Further machine learning algorithms filtered five biomarkers which have the potential to become diagnostic biomarkers for AF. Our 5-biomarkers model provides new ideas for diagnostic and treatment for AF.

### Limitation

Firstly, we have identified genes changes in 7-day rapid pacing model in this study, however, this model is reflecting atrial remodeling, but not directly by AF. Another limitation of this study is that our analysis is based solely on gene expression data, without direct assessment of the lactylation modifications. Given the emerging role of lactylation in regulating protein function, its potential impact on the five genes which we identified remains unclear. Future research incorporating proteomic approaches, such as mass spectrometry-based lactylation profiling, would be necessary to validate the functional significance of these modifications. Finally, while our study provides insights into the expression of lactylation-related genes, it is important to consider potential species differences in their regulation. Dogs and humans may exhibit distinct patterns of lactylation regulation due to differences in metabolic pathways, enzymatic activity, and epigenetic modifications. Future studies incorporating direct lactylation profiling, such as proteomic or metabolomic approaches, could provide a deeper understanding of species-specific lactylation dynamics and their relevance to atrial fibrillation.

## Data Availability

The original contributions presented in the study are included in the article/Supplementary Material, further inquiries can be directed to the corresponding authors.

## Supplementary material

Supplementary Figure S1 Removing the batch of three datasets of AF as training dataset.

Supplementary Figure S2 Enrichment analysis of co-expression modules. (A) Enrichment analysis of MEturquoise. (E) Enrichment analysis of MEblue.
