# Supplementary figures and images for: Comprehensive analysis of lactylation-related gene and immune microenvironment in atrial fibrillation
Source: Front Cardiovasc Med. 2025 Apr 22;12:1567310. doi: 10.3389/fcvm.2025.1567310 (PMC12053079; doi:10.3389/fcvm.2025.1567310)

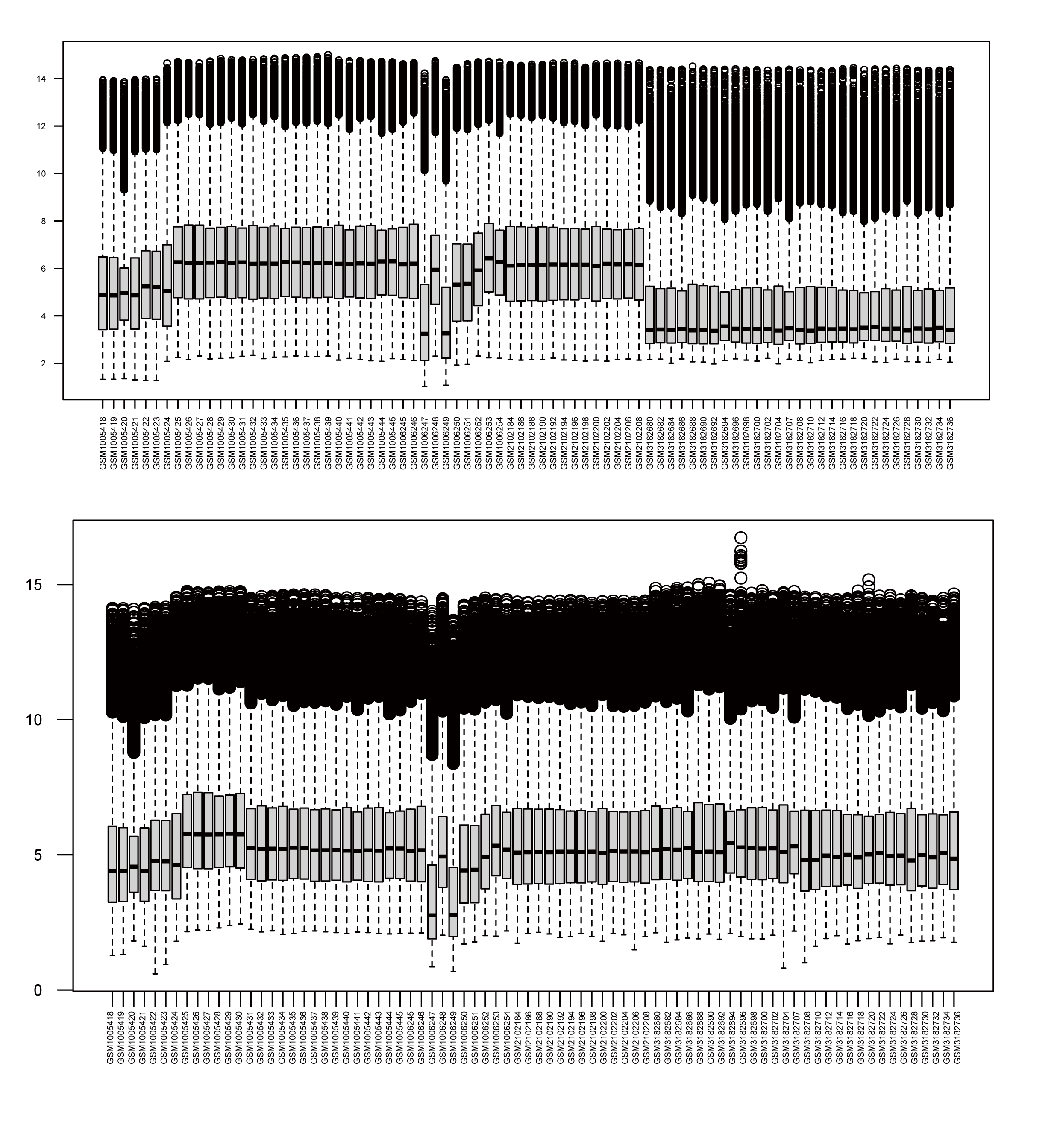

Supplement: Supplementary Figure S1 — Removing the batch of three datasets of AF as training dataset. [file Image1.tif]

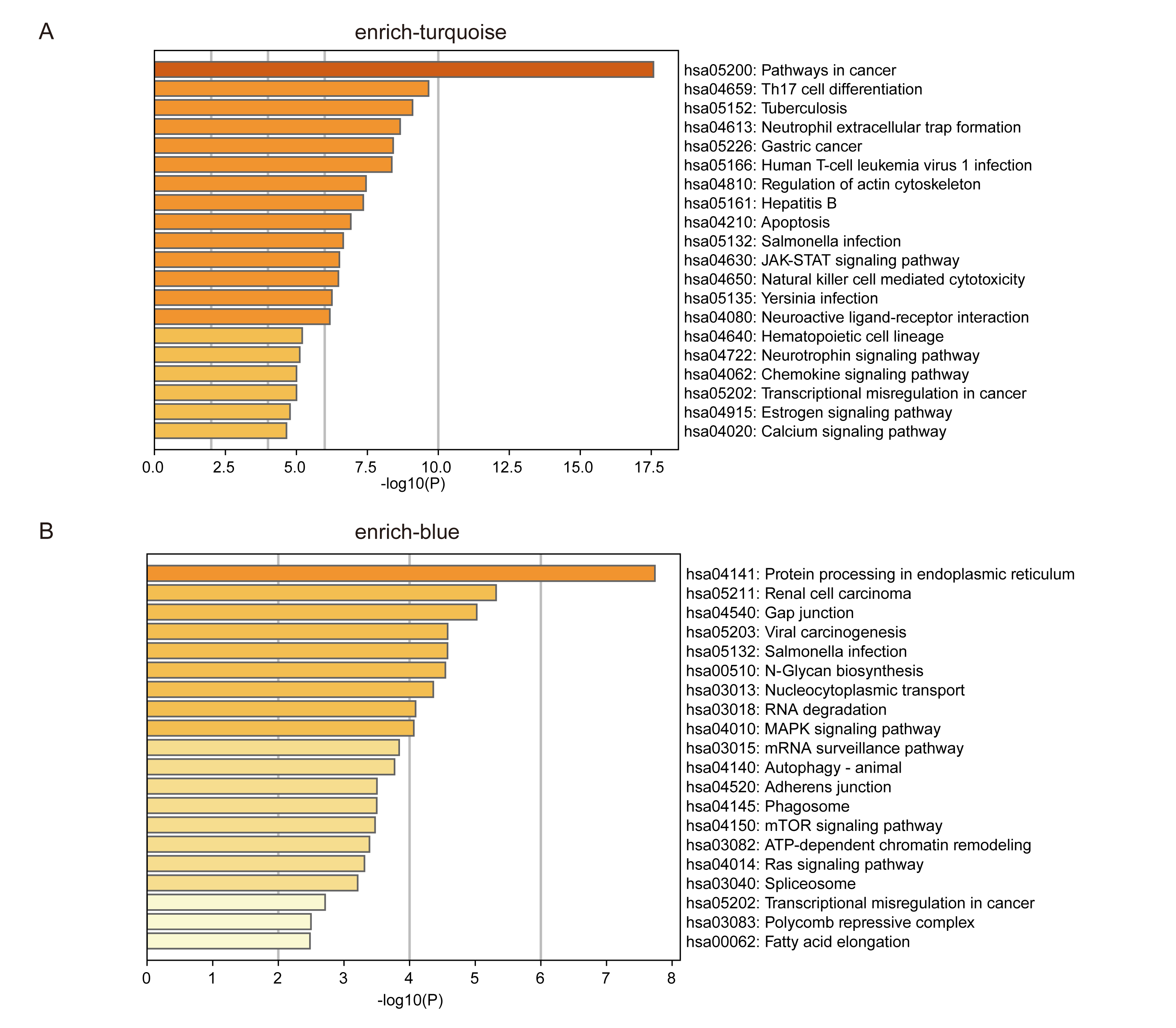

Supplement: Supplementary Figure S2 — Enrichment analysis of co-expression modules. (A) Enrichment analysis of MEturquoise. (E) Enrichment analysis of MEblue. [file Image2.tif]
